# Imbalance of thalamic metabolites in an experimental model of hypertension: role of bergamot polyphenols

**DOI:** 10.3389/fnint.2023.1271005

**Published:** 2023-09-14

**Authors:** Cristina Carresi, Antonio Cardamone, Anna Rita Coppoletta, Annachiara Mollace, Vincenzo Musolino, Micaela Gliozzi, Vincenzo Mollace

**Affiliations:** ^1^Veterinary Pharmacology Laboratory, Department of Health Sciences, Interregional Research Center for Food Safety and Health IRC-FSH, University “Magna Graecia” of Catanzaro, Catanzaro, Italy; ^2^Pharmacology Laboratory, Department of Health Sciences, Interregional Research Center for Food Safety and Health IRC-FSH, University “Magna Græcia” of Catanzaro, Catanzaro, Italy; ^3^Pharmaceutical Biology Laboratory, Department of Health Sciences, Interregional Research Center for Food Safety and Health IRC-FSH, University “Magna Græcia” of Catanzaro, Catanzaro, Italy

**Keywords:** hypertension, cerebral metabolites, thalamus, polyphenols, bergamot

## Abstract

Cerebral metabolites are associated with different physiological and pathological processes in brain tissue. Among them, the concentrations of *N*-acetylaspartate (NAA) and choline-containing compounds (Cho) in the thalamic region are recognized and analyzed as important predictive markers of brain impairment. The relationship among hypertension, modulation of brain metabolite levels and cerebral diseases is of recent investigation, leaving many unanswered questions regarding the origin and consequences of the metabolic damage caused in grey and white matter during hypertension. Here we provide evidence for the influence of hypertension on NAA and Cho ratios in hypertensive rat thalamus and how the use of natural occurring compounds ameliorates the balance of thalamic metabolites.

## 1. Introduction

Hypertension significantly contributes to the global burden of disease also emerging as a pathogenic factor in vascular-based cognitive impairment. Indeed, high blood pressure alters the structure and function of cerebral vasculature and represents the main risk factor for cerebral small vessel diseases and neuronal loss (Guenot et al., 2004; Khan et al., 2007). In fact, cerebral small vessel disease can cause silent ischemia in the perforating arteries of the thalamus as well as in the cortical vessels of the insula, leading to a long-term functional outcome characterized by a significantly increased risk of cognitive impairment (Norrving, 2003). The latter, triggered by high blood pressure, has also been associated with Alzheimer’s disease (Scheltens et al., 2016). However, the molecular mechanisms of detrimental cerebrovascular effects leading to cognitive impairment remain to be fully understood. Some studies have shown that the greatest harmful impact of hypertension mostly affects certain brain areas, such as those involved in cognition, e.g., hippocampus, medial thalamus, and frontal lobe, which undergo a reduction of resting cerebral blood flow, endothelial dysfunction, lipohyalinosis and fibrinoid necrosis leading to pathological remodeling, stiffening, vessel wall degeneration, and atherosclerosis of intracranial and extracranial cerebral arteries (Iadecola and Gottesman, 2019).

In particular, the failure of endothelium-dependent vasodilation represents a prelude to the onset of atherosclerosis and directly correlates with white matter lesion burden and microbleeds (Nezu et al., 2015). Thal and colleagues demonstrated that in the presence of mild or severe atherosclerosis, simultaneous involvement of small thalamic vessels can occur, as shown in autopsy brains (Thal et al., 2003). Furthermore, interesting studies using PET correlated a reduction in regional cerebral blood flow in the thalamus of elderly hypertensive brains with the decrease in certain cerebral metabolites, such as *N*-Acetylaspartate (NAA) creatine (Cr) ratio, as a consequence of hypoperfusion (Beason-Held et al., 2007; Salem et al., 2008). Indeed, spectroscopic detection of NAA in brain tissue allows for an accurate assessment of neuronal functional loss and is involved in brain energy metabolism (Singhal et al., 2017; Williamson et al., 2021). Furthermore, choline (Cho) levels are related to cell membrane integrity and inflammation and creatine (Cr) represents an important indicator of oxidative metabolism (Ross et al., 2006). Patients with severe hypertension show a reduced oxygen consumption in regions with reduced cerebral blood flow suggesting that, during disease progression, the vascular dysfunction and the reduced cerebral blood flow could be related to reduced metabolic demands due to brain dysfunction and damage (Fujishima et al., 1995). Chronic cerebral hypoperfusion is, therefore, associated with increased inflammatory state and oxidative stress (Liu et al., 2007), critical factors in the alterations of neurovascular and endothelial function produced by hypertension (Hamel et al., 2008).

In this perspective, the hypothesis of preventing or reducing the onset of oxidative stress using natural occurring antioxidant compounds could be of particular importance for the management of hypertension-induced cerebral complications.

We now report that a polyphenol-rich fraction of citrus bergamot (BPF), characterized by peculiar anti-oxidative and anti-inflammatory effects, already observed in different experimental animal models and clinical trials (Carresi et al., 2018, 2020; Musolino et al., 2020; Mollace et al., 2021), ameliorates the total NAA (tNAA)/total Cr (tCr) and total Cho (tCho)/tCr ratios in the thalamus of hypertensive rats.

## 2. Materials and methods

### 2.1. BPF preparation

*Citrus bergamia* Risso and Poiteau fruits were harvested in a coastal area between Bianco and Reggio Calabria, Italy. Bergamot possesses a profile of flavonoids and glycosides which can be considered unique in its various forms (essential oil, hydro-alcoholic extract, and fruit juice) differing from other citrus fruits for the composition of its flavonoids and also for their particularly high juice content (Gardana et al., 2008; Salerno et al., 2016). The extraction procedure of the bergamot polyphenolic fraction (BPF), provided by Herbal and Antioxidant Derivatives (H&AD) (Bianco, Reggio Calabria, Italy), was performed as previously reported (Carresi et al., 2018). Briefly, bergamot juice was obtained from peeled-off fruits by squeezing. The juice was oil fraction-depleted by stripping, clarified by ultra-filtration and loaded on to a suitable polystyrene resin column able to absorb polyphenol compounds of molecular weight between 300 and 600 Da (Mitsubishi). Polyphenol fractions were eluted by a 1 mM KOH solution. The basic eluate was incubated at a rocking platform to reduce the furocumarin content. Next, the derived phytocomplex was neutralized by filtration on cationic resin at acidic pH. Finally, it was vacuum dried and minced to the desired particle size to obtain BPF powder. BPF powder was analyzed for flavonoid, furocoumarin and other polyphenols by UHPLC-HRMS/MS and Orbitrap spectrometer (Salerno et al., 2016). The method allows to reveal the presence of the main flavonoids neoeriocitrin, naringin, neohesperidin but also of all HMG-family such as bruteridin, melitidin with other HMG species and also the flavonoids 6,8-di-C-glicosides.

### 2.2. Study design

Wistar male rats (*n* = 40) at 8–10 weeks of age (body weight 400 ± 10 g) were housed under standard laboratory conditions. Hypertension was induced by left renal artery ligation (RAL). Briefly, the animals were anaesthetized with 5% isoflurane in oxygen and then maintained with 2% isoflurane in oxygen and monitored constantly during the surgery. A vertical incision was made in the midline of the back, fat was removed from the renal pelvis, and the kidney was exposed. Subsequently, the right renal artery was identified and exposed, and the vessel was ligated tightly with 4.0 suture. Finally, the kidney was repositioned *in situ* and the incision closed. The rats undergoing surgery were then housed individually in cages. After complete recovery from surgery (approximatively 48 h after) the animals were treated with deoxycorticosterone acetate (DOCA) (20 mg/kg, s.c.), twice a week for 4 weeks, and 1% sodium chloride (NaCl) water (*n* = 10). Hereafter, animals treated as described above are referred as RAL DOCA-Salt. A subgroup of hypertensive rats received BPF, dissolved in drinking water (100 mg/kg/day for 28 consecutive days, *n* = 10) by gavage. A control group was treated with sub-cutaneous injection of saline (*n* = 8, CTRL). At baseline, after 2 weeks and at the end of the experimental period, all animals underwent nuclear magnetic resonance (NMR) and spectroscopy. Finally, the animals were sacrificed with an overdose of sodium pentobarbital (150 mg/kg, i.p.). All the experimental procedures conducted on animals were performed according to protocols approved by the Animal Care of University Magna Graecia of Catanzaro, in accordance with the guidelines of the European Commission for the care of animals used for scientific purposes (Directive 2010/63/EU).

### 2.3. ^1^H-MRS of the thalamus

Prior to proton magnetic resonance spectroscopy (^1^H-MRS) acquisition, rats were anesthetized with 4% isoflurane (Forane, Abbott) vaporized in O_2_ (flow rate: 2 L/min). During the acquisition, anesthesia was maintained at around 2%, respiratory rate between 40 and 60 breaths per minute, body temperature at 37°C. ^1^H-MRS spectra were acquired with a Bruker Pharmascan MR scanner at 7 Tesla (Bruker Biospin MRI GmbH, Ettlingen, Germany). The voxel, within which spectroscopy was performed, was precisely positioned in the thalamus (VOI 3 × 3 × 3 mm) according to anatomical landmarks, based on a rat brain atlas. After voxel placement, spectra with and without water suppression were acquired, which are required for absolute quantification of metabolites within the voxel. This quantification was performed using TARQUIN 4.3.10 software as previously described (Nucera et al., 2022).

### 2.4. Statistical analysis

Data were analyzed with GraphPad Prism software version 9.1.1 of 2021 (GraphPad Software, San Diego, CA, United States). Results are shown as mean ± SEM. Bartlett’s test was performed on each dataset to ensure that the variance of the sets was homogeneous. Normally distributed data were analyzed by one way ANOVA followed by Tukey’s or Bonferroni’s test for multiple comparisons. While data without normal distribution were analyzed using Kruskal–Wallis analysis of variance and subsequent Dunn’s tests. Student’s t-test and Mann–Whitney test were used, respectively, to compare data from two specific groups having a normal or non-normal distribution. For comparison of groups of categorical values, Pearson’s chi-square test was performed with statistical software (Stat Soft, Tulsa, OK, United States).

## 3. Results

### 3.1. BPF ameliorates tNAA/tCr and tCho/tCr ratios in the thalamus of RAL DOCA-Salt rats

**Figure 1:**
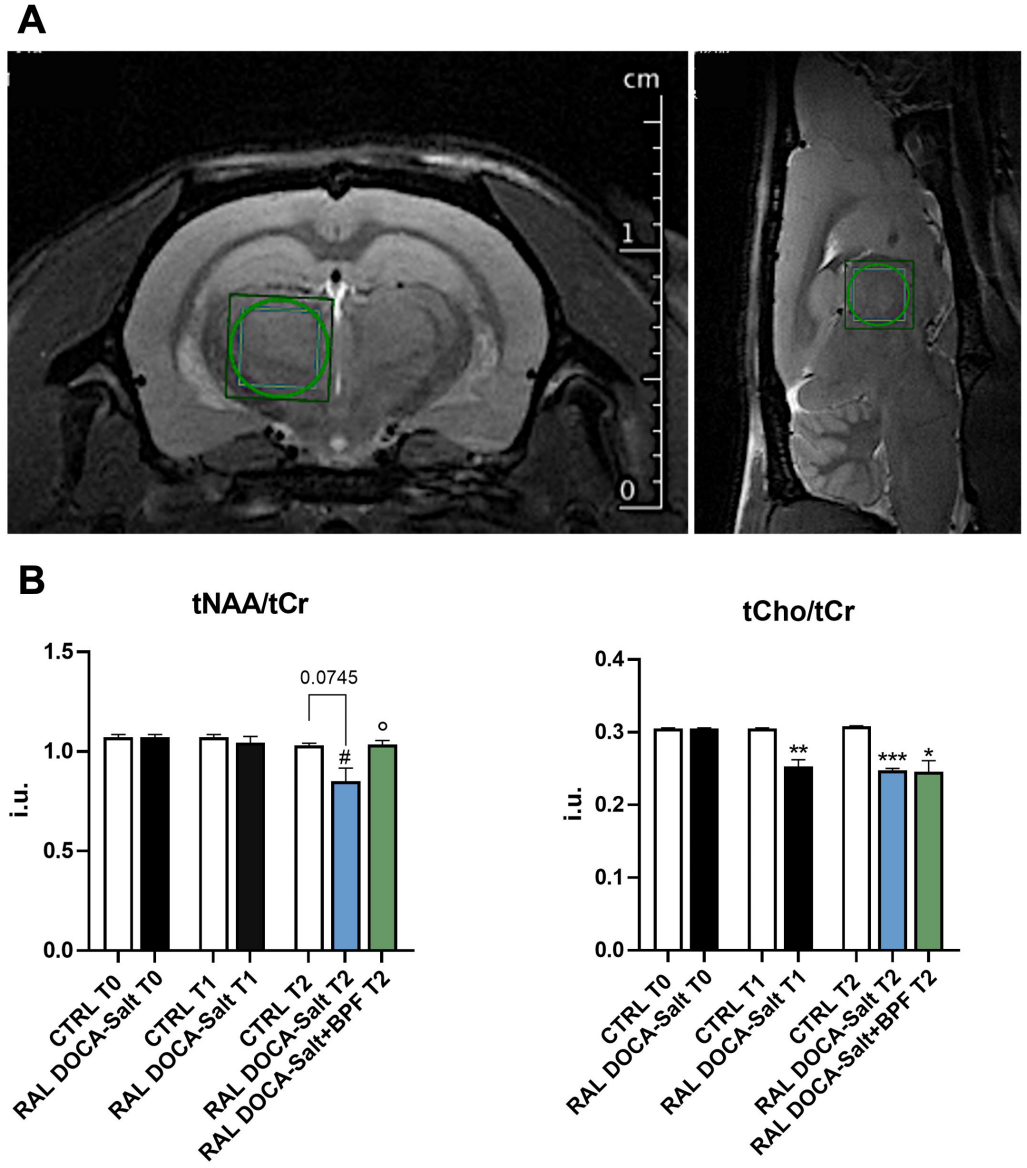
^1^H-MRS of the rat thalamus. **(A)** Representative T2_turboRARE weighted images in axial and sagittal view of rat brain and corresponding voxel localized in the thalamus (3 × 3 × 3 mm^3^). **(B)** Histograms showing the modulation of the tNAA/tCr ratio and of the tCho/tCr in the different experimental groups. The data are presented as mean ± SEM.; i.u., institutional units. ^#^*p* < 0.05 vs. RAL DOCA-Salt T1, °*p* < 0.05 vs. RAL DOCA-Salt T2, *p < 0.05 vs. CTRL T2, **p < 0.01 vs. CTRL T1, ***p < 0.001 vs. CTRL T2.

The *^1^H-MRS* spectra quantification analysis in the thalamus showed no differences between groups for both total-N-AcetylAspartate/totalCreatine (tNAA/tCr) and totalCholine/totalCreatine (tCho/tCr) ratios at time 0 (Figures 1A,B). A reduction tending to significance of the tNAA/tCr ratio, emerged in the thalamus of RAL DOCA-Salt rats compared to the thalamus of CTRL at the final time point (T2) (0.85 ± 0.13 i.u. vs. 1.03 ± 0.02 i.u. in CTRL T2, *p* = 0.0745, Figure 1A). A significant reduction of the tNAA/tCr ratio was observed in the group of RAL DOCA-Salt rats at the T2 compared to the same animals at the intermediate time point (T1) (0.85 ± 0.13 i.u. vs. 1.04 ± 0.08 i.u. in RAL DOCA-Salt T1, ^#^*p* < 0.05, Figure 1A). Interestingly, a significant increase in the tNAA/tCr ratio was observed in the group of RAL DOCA-Salt rats treated with BPF compared to the RAL DOCA-Salt rats treated with vehicle at the T2 (1.036 ± 0.042 i.u. vs. 0.85 ± 0.13 i.u. in RAL DOCA-Salt T2, °*p* < 0.05, Figure 1A). In addition, a statistically significant reduction of the tCho/tCr ratio was highlighted, in the RAL DOCA-Salt animals and in the BPF treated ones, compared to the CTRL rats at the T2 (0.24 ± 0.005 i.u. and 0.024 ± 0.034 i.u. vs. 0.30 ± 0.001 i.u. in CTRL T2, respectively, ***p < 0.001 and *p < 0.05, Figure 1B). The statistically significant reduction of the tCho/tCr ratio in the thalamus of RAL DOCA-Salt rats compared to the thalamus of CTRL rats was already recorded starting from the T1 (0.25 ± 0.026 i.u. vs. 0.30 ± 0.001 i.u. in CTRL T1, **p < 0.01, Figure 1B).

## 4. Discussion

The brain is among the organs that could be further damaged by increased blood pressure.

Interesting epidemiological studies in elderly and midlife patients clearly shown that the main risk factors for cardiovascular and cerebrovascular diseases such as hypertension or carotid arteriosclerosis, are associated with increased incidence of cognitive impairment, dementia, Alzheimer’s disease (Tzourio et al., 1999; Hachinski and Munoz, 2000).

In a subgroup of patients belonging to a large population-based study, the association between the presence of hypertension and low levels of tNAA/tCr in specific brain areas such as the thalamus and the insular cortex was identified in 65% of the patients (3C Study Group, 2003; Khan et al., 2007).

In has been also demonstrated that hypertension is capable of inducing damage to the cerebral arteries and collateral branches causing cerebral hypoperfusion and electrolyte imbalance. Such electrolyte imbalance often leads to a metabolic disturbance which can be identified in clinical practice through the quantification of tNAA/tCr and tCho/tCr ratios (Salem et al., 2008; Suri et al., 2017).

Indeed, in our experimental model of cardio-renal hypertension, the quantification analysis of the thalamic spectra, based on magnetic resonance proton spectroscopy (^1^H-MRS), allowed to reveal a significant reduction in tNAA/tCr and tCho/tCr ratios in RAL DOCA-Salt rats compared to control ones.

Based on these experimental data, which agree with clinical data present in the literature, we hypothesize that the reason for the decrease in cerebral metabolite levels could be traced back to the electrolyte imbalance caused by hypertension, and in particular, by the reduction of cerebral blood flow through the small perforating and cortical arteries.

Indeed, Ben Salem and colleagues previously demonstrated the influence of hypertension on tNAA/tCr and tCho/tCr ratios in brain tissues of an elderly population. The proton magnetic resonance spectroscopy (MRS) results showed that in the insula and thalamus of hypertensive patients, the tNAA/tCr ratios were significantly lower than in normotensive ones. In addition, tNAA/tCr and tCho/tCr ratios were significantly correlated with Trail Making Test (TMT) B-A scores at the thalamus, insula and periventricular white matter levels (Salem et al., 2008). Interestingly, from the spectroscopic analysis of the metabolites considered in our experiments, a significant increase in the tNAA/tCr ratio was highlighted in rats treated with BPF compared to hypertensive rats treated with vehicle alone. Recent scientific evidence supports the result obtained, indicating how some polyphenols deriving from green tea extraction improve chronic cerebral hypoperfusion through the modulation of oxidative stress (Xu et al., 2010). Oxidative stress plays an important role in the pathogenesis of several neurodegenerative diseases by interfering with cognitive decline in the initial stages of these diseases (Hamel et al., 2008). Furthermore, a permanent global hypoperfusion condition can lead to increased oxidation reducing antioxidant capacity (Liu et al., 2007). Therefore, the chance of intervening by blocking the response to oxidative stress associated with chronic cerebral hypoperfusion may be important for the management of cognitive impairment or dementia characteristic of certain cardiovascular and cerebrovascular diseases.

## 5. Conclusion

Such evidence of the influence of hypertension on tNAA/tCr and tCho/tCr ratios could better explain the relationship between brain structure and modulation of some metabolites, providing new insights into hypertension-induced brain complications and the contribution that the use of natural compounds could give to their management.

## Data availability statement

The raw data supporting the conclusions of this article will be made available by the authors, without undue reservation.

## Ethics statement

The animal study was approved by the Animal Care of University Magna Graecia of Catanzaro, in accordance with the guidelines of the European Commission for the care of animals used for scientific purposes (Directive 2010/63/EU). The study was conducted in accordance with the local legislation and institutional requirements.

## Author contributions

CC: Conceptualization, Data curation, Formal Analysis, Investigation, Methodology, Supervision, Visualization, Writing – original draft, Writing – review & editing. AC: Data curation, Formal Analysis, Investigation, Methodology, Software, Writing – original draft. ARC: Data curation, Investigation, Methodology, Writing – original draft. AM: Data curation, Investigation, Writing – original draft. VMu: Conceptualization, Data curation, Formal Analysis, Methodology, Supervision, Visualization, Writing – review & editing. MG: Conceptualization, Data curation, Methodology, Project administration, Supervision, Writing – review & editing. VMo: Funding acquisition, Project administration, Resources, Supervision, Validation, Visualization, Writing – review & editing.

## Funding

The author(s) declare financial support was received for the research, authorship, and/or publication of this article. This work has been supported by the public resources from the Italian Ministry of Research, PON-MIUR 03PE000_78_1, PONMIUR 03PE000_78_2; POR Calabria FESR FSE 2014–2020 Asse 12-Azioni 10.5.6 e 10.5.12. POR Calabria FESR FSE 2014–2020 azione 1.5.1: progetto AgrInfra Calabria.

## Conflict of interest

The authors declare that the research was conducted in the absence of any commercial or financial relationships that could be construed as a potential conflict of interest.

## Publisher’s note

All claims expressed in this article are solely those of the authors and do not necessarily represent those of their affiliated organizations, or those of the publisher, the editors and the reviewers. Any product that may be evaluated in this article, or claim that may be made by its manufacturer, is not guaranteed or endorsed by the publisher.
